# Adjuvant neuroprotective therapies for acute ischemic stroke in the reperfusion era: a comparative narrative review of clinical evidence for edaravone, edaravone-dexborneol, and butylphthalide

**DOI:** 10.3389/fneur.2026.1899933

**Published:** 2026-08-12

**Authors:** Weiqing Wang, Junsen Ye

**Affiliations:** 1 Department of Neurology, First People's Hospital of Jiande City, Hangzhou, Zhejiang, China; 2 Department of Scientific Research, First People's Hospital of Jiande City, Hangzhou, Zhejiang, China

**Keywords:** acute ischemic stroke, adjunctive therapy, butylphthalide, edaravone-dexborneol, endovascular thrombectomy, neuroprotection, reperfusion injury

## Abstract

Reperfusion therapies, including intravenous thrombolysis and endovascular treatment (EVT), are central to acute ischemic stroke (AIS) management; however, ischemia–reperfusion injury may limit recovery despite successful recanalization. This structured narrative review compares the clinical evidence, safety signals, and mechanistic literature for edaravone, butylphthalide (NBP), and the fixed-dose combination edaravone-dexborneol (EDB) as adjunctive neuroprotective approaches. The evidence base is heterogeneous with respect to population, background reperfusion treatment, outcome timing, and geographical setting. In TASTE-2, EDB administered before EVT was associated with a modest increase in 90-day functional independence versus placebo (55.0% vs. 49.6%, *p* = 0.05), without an apparent safety signal; bridging alteplase was permitted in a subset of participants. In the BAST trial, NBP improved the prespecified 90-day functional outcome versus placebo among patients receiving intravenous thrombolysis and/or EVT (56.7% vs. 44.0%). Observational studies and meta-analyses provide supportive but non-confirmatory evidence for edaravone. Network meta-analyses generate relative rankings at individual endpoints, but indirect comparisons and differing outcome networks preclude definitive between-agent selection. Mechanistic studies indicate overlapping antioxidant, anti-inflammatory, mitochondrial, and neurovascular effects rather than exclusive pathway regulation. Biomarker and imaging approaches, including exploratory GFAP and UCH-L1 measurements, are promising research tools but are not yet validated for treatment selection in AIS. Accordingly, these agents should be regarded as candidates for further evidence-informed adjunctive use; future trials are needed to define reproducible patient-selection and treatment-timing strategies.

## 1 Introduction

Acute ischemic stroke (AIS) is a neurological emergency associated with high incidence, disability, and mortality and remains a major global public-health challenge. In recent years, reperfusion therapies, including intravenous thrombolysis and endovascular treatment (EVT), have become central to acute-phase AIS management and have improved outcomes (1, 2). Nevertheless, some patients have suboptimal neurological recovery despite successful vascular recanalization, a phenomenon closely linked to ischemia–reperfusion injury (IRI) (1). Although reperfusion restores cerebral blood flow, it can trigger oxidative stress, neuroinflammation, and blood–brain barrier disruption, thereby exacerbating secondary brain injury (1, 3). Accordingly, adjunctive neuroprotective strategies that preserve neurons in the ischemic penumbra remain an important research priority (4, 5). Edaravone, butylphthalide (NBP), and the fixed-dose combination edaravone-dexborneol (EDB) have distinct pharmacological profiles and an evolving clinical evidence base (4, 6). Edaravone is a free-radical scavenger with longstanding clinical use; NBP has been linked to effects on microcirculation and mitochondrial function; and EDB combines edaravone with dexborneol to target oxidative stress and inflammatory pathways. This review compares the clinical efficacy and safety evidence for these agents in the reperfusion era, summarizes their overlapping mechanisms, considers context-specific applications, and outlines priorities for future neuroprotective drug development in AIS.

## 2 Review type, search strategy, and evidence appraisal

This article is a structured narrative review, not a systematic or scoping review. Searches were conducted in PubMed/MEDLINE, Embase, Web of Science Core Collection, Scopus, the Cochrane Library, and Google Scholar from database inception to 31 May 2026. The strategy combined AIS/reperfusion terms with drug-specific terms and was supplemented by backward and forward citation tracking of pivotal trials and relevant reviews. Detailed database-specific search strings and Boolean operators are provided in Appendix 1. Searches were limited to English-language publications with sufficient full-text methodological information for appraisal; no study-design restriction was applied at the search stage.

Eligible studies included randomized controlled trials, systematic reviews and meta-analyses, large observational cohorts, and mechanistic preclinical or translational biomarker studies relevant to edaravone, EDB, or NBP in AIS. We prioritized studies reporting functional outcomes, safety outcomes, biological mechanisms, or biomarker-guided stratification, particularly where patients received contemporary reperfusion therapy. We excluded duplicate reports, case reports, editorials, conference abstracts without sufficient peer-reviewed detail, and studies in non-ischemic neurological conditions unless they provided directly relevant mechanistic or biomarker context. Titles and abstracts were screened against these criteria, followed by full-text appraisal of potentially relevant reports. Because this was a narrative review, no PRISMA flow diagram or pooled meta-analysis was undertaken.

Because this was not designed as a systematic review, we did not apply a formal risk-of-bias instrument to every included study. Evidence was instead interpreted according to study design, randomization and blinding, sample size, outcome timing and relevance, adjustment for confounding, consistency, and major limitations. Randomized trials provide the most direct evidence for the intervention and population studied, registry studies are susceptible to residual confounding, and animal, cellular, and biomarker studies are mechanistic or hypothesis-generating. Table 1 summarizes these evidence categories and their principal limitations. This approach does not replace a formal risk-of-bias assessment and should not be interpreted as a quantitative grading of certainty.

**Table 1:** Summary of major clinical evidence for adjunctive neuroprotective agents in acute ischemic stroke.

Evidence type/study	Agent	Population/sample	Intervention context	Primary outcomes	Key limitations
TASTE-2 RCT	Edaravone-dexborneol (EDB)	EVT-treated AIS, 1,360 in ITT analysis	EDB initiated before thrombectomy, bridging alteplase permitted in a subset	90-day mRS 0–2: 55.0% vs. 49.6% (RR 1.11, 95% CI 1.00–1.23, *p* = 0.05)	Context-specific EVT population, borderline result, subgroup findings exploratory
BAST RCT	NBP	1,216 AIS patients receiving IVT and/or EVT	Adjunct to contemporary reperfusion therapy	90-day favorable outcome: 56.7% vs. 44.0%	One trial population, optimal timing and subgroup benefit remain uncertain
Japanese national database study	Edaravone	>11,000 EVT-treated AIS patients	Early edaravone use	Associated with better discharge outcomes and lower in-hospital mortality/ICH	Observational, residual confounding, discharge rather than 90-day outcome
Fidalgo et al. meta-analysis	Edaravone	19 studies, predominantly Asian and mainly observational	Acute ischemic stroke	Pooled association with better 90-day outcomes and lower mortality	High heterogeneity, limited direct evidence in contemporary reperfusion settings
Wang et al. network meta-analysis	Multiple agents	42 RCTs, 12,210 participants	Indirect comparison across endpoint-specific networks	SUCRA rankings varied by 7-, 14-, and 90-day outcomes	Rankings do not prove superiority, endpoint networks differed
Dang et al. Bayesian NMA	Multiple agents plus rt-PA	70 RCTs, 8,152 participants	Neuroprotective agents combined with thrombolysis	Mostly 2-week NIHSS and Barthel Index comparisons, EDB, edaravone, and NBP combinations included	Indirect comparisons, trial heterogeneity, and ranking-based inference

## 3 Clinical efficacy and safety evidence

### 3.1 EDB: direct clinical evidence and limitations

EDB is a fixed-dose combination of edaravone and dexborneol. In TASTE-2, patients with anterior-circulation large-vessel occlusion were randomized to EDB or placebo before EVT; bridging alteplase was permitted but was not the defining treatment context (7, 8). In the modified intention-to-treat analysis, 55.0% of the EDB group and 49.6% of the placebo group achieved functional independence (mRS 0–2) at 90 days (risk ratio 1.11, 95% CI 1.00–1.23, *p* = 0.05). The result supports a possible adjunctive role for EDB in the studied EVT population, but the borderline statistical result, context-specific population, and exploratory subgroup findings warrant caution. Two phase III trials provide additional evidence for EDB in broader AIS populations. In TASTE, 1,165 patients treated within 48 h were randomized to intravenous EDB or edaravone; the proportion with mRS 0–1 at 90 days was higher with EDB (67.18% vs. 58.97%; odds ratio 1.42, 95% CI 1.12–1.81; *p* = 0.004) (9). In TASTE-SL, 914 patients treated within 48 h were randomized to sublingual EDB or placebo; mRS 0–1 at 90 days occurred in 64.4 and 54.7% of patients, respectively (risk difference 9.70, 95% CI 3.37–16.03%; *p* = 0.003), with similar adverse-event rates (10). These trials were not designed to establish efficacy in contemporary EVT-treated populations; notably, TASTE-SL excluded endovascular therapy. Their findings therefore broaden the clinical evidence for EDB in selected AIS populations but should not be extrapolated directly to peri-EVT use. Meta-analyses of EDB trials also report favorable functional and hemorrhagic-transformation outcomes, but their conclusions are constrained by heterogeneous trial designs and settings (11, 12). A posterior-circulation retrospective study of EDB in 101 patients and should be interpreted as association-based evidence (13). Similarly, the phase II trial after successful thrombectomy evaluated EDB (*n* = 200) and did not show a statistically significant difference in its primary 90-day functional endpoint (14). Together, these studies do not establish uniform benefit across all reperfusion-treated patients (Table 1).

### 3.2 NBP: clinical evidence and limitations

NBP has been evaluated as an adjunct to contemporary stroke care. In the BAST trial, 1,216 patients receiving intravenous thrombolysis and/or EVT were randomized to NBP or placebo (15, 16). A favorable 90-day functional outcome occurred more often with NBP (56.7% vs. 44.0%; odds ratio 1.70, 95% CI 1.35–2.14), without an observed increase in serious adverse events (16). This trial provides important direct evidence in the studied population, but it does not establish NBP as a universal or preferred component of reperfusion therapy. Generalizability, optimal timing, and differential benefit across reperfusion subgroups require confirmation.

Observational studies have reported that NBP use during EVT was associated with better 90-day functional outcomes and lower mortality without an excess of symptomatic intracranial hemorrhage (17, 18). These reports are supportive, but residual confounding, treatment-selection bias, and differences in background care mean that they cannot establish causality or replace randomized comparisons in EVT-specific populations.

Studies of NBP administered with intravenous thrombolysis evaluate an adjunctive regimen rather than pharmacological or statistical synergy. A preliminary clinical study comparing alteplase alone with alteplase plus NBP reported changes in cerebral circulation, coagulation-related measures, and neurological recovery (19). An additional study associated early NBP administration after thrombolysis with a lower risk of reocclusion (20). These findings should be regarded as preliminary and require larger blinded trials with prespecified clinical endpoints.

The phase II EXIT-BT study examined tenecteplase plus NBP in an extended time window and reported early safety and neurological findings (21). Its size and phase II design preclude definitive conclusions about clinical efficacy. Overall, available NBP evidence supports further evaluation as an adjunctive therapy rather than routine use in all reperfusion settings.

### 3.3 Edaravone: clinical evidence and limitations

Edaravone is a clinically used free-radical scavenger, but evidence for its benefit in the reperfusion era remains heterogeneous. A systematic review and meta-analysis by Fidalgo et al. reported associations with improved 90-day functional outcomes and lower mortality without increased intracranial hemorrhage (22). However, most included primary studies were observational, were conducted in Asian populations, particularly in Japan, and showed high heterogeneity; additional randomized evidence outside these settings is needed. Consequently, these pooled estimates should not be interpreted as definitive proof of efficacy in contemporary reperfusion-treated populations.

In a large Japanese administrative database study of EVT-treated patients, early edaravone use was associated with higher functional independence at discharge, lower in-hospital mortality, and less intracranial hemorrhage (23). The observational design leaves residual confounding possible. For intravenous thrombolysis, a meta-analysis of predominantly small trials suggested better short-term neurological outcomes and less intracranial hemorrhage with edaravone plus alteplase, whereas a Japanese propensity-matched cohort found an association with lower disability at discharge (24, 25). These findings describe adjunctive-treatment associations; they do not demonstrate clinical synergy or establish edaravone as a routine component of thrombolysis.

Preclinical studies provide mechanistic support for edaravone’s antioxidant and mitochondrial effects, but these results should be distinguished from clinical efficacy evidence. Observational and experimental findings may help define hypotheses for future trials, including whether baseline metabolic risk, infarct characteristics, or reperfusion status modify response. They do not justify claims that edaravone is indispensable or superior to other adjunctive approaches in current practice.

### 3.4 Network meta-analysis: comparative rankings and limitations

Network meta-analysis (NMA) can compare multiple interventions, but its findings require cautious interpretation. Wang et al. (26) synthesized 42 randomized controlled trials involving 12,210 patients with AIS and reported relative rankings for individual neurological and functional outcomes. The 7-day and 90-day outcomes were derived from different trial networks, and SUCRA rankings do not establish statistically significant superiority or stage-specific efficacy. A separate Bayesian NMA by Dang et al. (27) assessed neuroprotective regimens combined with intravenous thrombolysis across 70 RCTs enrolling 8,152 patients. The analysis included EDB plus rt-PA, edaravone plus rt-PA, and NBP plus rt-PA; outcomes were assessed mainly at 2 weeks and conclusions relied substantially on indirect comparisons and SUCRA rankings (27). These NMAs are useful for hypothesis generation, but they do not support preferential selection of NBP for long-term recovery or edaravone for acute-phase neuroprotection. Direct comparative trials with harmonized endpoints are needed before between-agent treatment selection can be recommended.

## 4 Diverse mechanisms of neuroprotection

### 4.1 Antioxidant and anti-inflammatory mechanisms: overlapping evidence

Oxidative stress and inflammatory cascades are important components of ischemia–reperfusion injury (1, 3). Edaravone, EDB, and NBP have each been linked to antioxidant and anti-inflammatory effects in experimental or clinical studies, but these mechanisms overlap across agents and are not exclusive explanations for clinical outcomes.

Edaravone is commonly described as a free-radical scavenger that can reduce reactive oxygen and nitrogen species in experimental ischemia–reperfusion injury. Experimental and review literature also supports effects on apoptosis-related and mitochondrial pathways, although translation from preclinical models to clinical benefit remains uncertain (28–30).

EDB combines edaravone with dexborneol. Preclinical and translational studies suggest that the combination may influence oxidative stress, inflammatory signaling, and blood–brain barrier-related injury, but these pathways are not exclusive to EDB and should not be equated with clinical efficacy (31–33).

NBP has also been associated with antioxidant and anti-inflammatory effects in experimental models. For example, Zhang et al. reported reduced pro-inflammatory mediators alongside changes in NF-kB/iNOS and Keap1/Nrf2-related signaling in a post-stroke cognitive-impairment model (34). These experimental findings do not demonstrate a drug-specific pathway or clinical synergy.

In animal models, DL-3-n-butylphthalide (NBP) combined with edaravone has been associated with changes in oxidative-stress, inflammatory, and apoptosis-related markers (35). These preclinical findings should be described as mechanistic observations rather than proof of clinical benefit from the combination.

### 4.2 Mechanism-associated pathways with overlapping evidence

The agents reviewed here have been linked to several signaling pathways, but the available literature does not support their classification as unique or drug-exclusive mechanisms. The cGAS-STING/STX17/autophagy and CXCL13/CXCR5/NF-kB studies cited here evaluated EDB (36, 37). In experimental models, NBP has been associated with angiogenesis-related signaling, including Sonic Hedgehog and HIF-1alpha/VEGF/Notch/Dll4, as well as endothelial mitochondrial homeostasis and antioxidant responses (34, 38–40). These pathway-level findings are hypothesis-generating, may overlap across agents and injury models, and should not be used to infer agent-specific clinical efficacy.

### 4.3 Intervention in multiple pathological mechanisms, including mitochondrial function, autophagy, and apoptosis

Mitochondrial dysfunction, altered autophagy, and apoptosis are common features of ischemia–reperfusion injury. In experimental stroke models, NBP has been linked to inhibition of mitochondrial Omi/HtrA2-mediated apoptosis (41). Edaravone-related studies suggest mitochondrial effects, including reduced mitochondrial oxidative stress and modulation of permeability-transition pathways (29, 30, 42). Evidence from nanoparticles or cardiac models should not be attributed to NBP in cerebral ischemia without direct supporting data. These mechanisms provide a biological rationale but remain distinct from proof of clinical benefit.

More recent edaravone-focused literature indicates that mitochondrial protection may be an important component of its cytoprotective profile, rather than merely a downstream consequence of free-radical scavenging. Experimental work suggests that edaravone can preserve mitochondrial structure and function, stabilize mitochondrial membrane potential, reduce mitochondrial ROS generation, support ATP production, inhibit opening of the mitochondrial permeability transition pore, and attenuate mitochondria-mediated apoptosis after ischemic or ischemia–reperfusion injury (29, 30). These mitochondrial effects are consistent with broader edaravone literature from the Kobeissy group and collaborators, which emphasizes oxidative stress, mitochondrial dysfunction, and multi-compartment biomarker responses across acute neurological injury models, including TBI and stroke-relevant reperfusion injury (43, 44). Edaravone may therefore have mitochondrial-stabilizing antioxidant effects in addition to its free-radical-scavenging activity.

Autophagy is context-dependent in cerebral ischemia. The cited cGAS-STING/autophagy study evaluated EDB and reported restored STX17-mediated autophagic flux with reduced inflammasome activation in experimental ischemia–reperfusion injury (36). Conversely, an NBP derivative (XY03-EA), rather than NBP itself, was reported to protect experimental animals while suppressing autophagy (45). These studies illustrate context-dependent autophagy modulation and should not be generalized to edaravone monotherapy or clinical treatment decisions.

Apoptosis is a convergent pathway of neuronal loss after ischemia. DL-3-n-butylphthalide (NBP) was reported to inhibit mitochondrial Omi/HtrA2-mediated apoptosis in an experimental model (41), and NBP plus edaravone altered apoptosis-related markers in mice (35). These findings remain preclinical and do not establish a clinically meaningful combination effect.

## 5 Application strategies and supporting evidence in specific clinical settings

### 5.1 Precision medicine and biomarker-guided neuroprotection

The heterogeneous response to adjunctive neuroprotective therapy suggests that future trials should move beyond a one-size-fits-all model and incorporate biologically informed patient selection. Candidate stratification variables include reperfusion status, collateral and perfusion-imaging mismatch, infarct core volume, hemorrhagic-transformation risk, age, diabetes or hyperglycemia, baseline oxidative-stress burden, and circulating markers of astroglial or neuronal injury (Table 2). This approach may help identify patients in whom oxidative stress, mitochondrial dysfunction, blood–brain barrier injury, or neuroinflammation is dominant and therefore more likely to respond to edaravone, EDB, or NBP.

**Table 2:** Potential biomarker and imaging framework for precision neuroprotection in AIS.

Stratification domain	Candidate marker	Biological meaning	Potential use	Current limitation
Astroglial injury	GFAP	Astrocytic injury and blood–brain barrier disruption	Exploratory tissue-injury or hemorrhagic-risk endpoint	Not validated for AIS treatment selection
Neuronal injury	UCH-L1	Neuronal cell-body injury	Exploratory pharmacodynamic neuronal-injury endpoint	Kinetics and cutoffs in AIS require validation
Oxidative stress	MDA, 4-HNE, 8-OHdG, nitrotyrosine	Reactive oxygen and nitrogen species burden	Mechanism-linked enrichment hypothesis	Assay standardization and clinical thresholds remain uncertain
Mitochondrial injury	ATP-related metabolites, mitochondrial DNA, lactate/pyruvate	Energy failure and mitochondrial damage	Mechanistic endpoint in early-phase studies	Limited prospective AIS trial evidence
Imaging phenotype	Core-penumbra mismatch, collateral grade, reperfusion status	Viable tissue and reperfusion-injury risk	Prospective selection variable for trial testing	Requires harmonized imaging protocols

The TBI biomarker field provides a useful translational framework. GFAP and UCH-L1 have become clinically important blood biomarkers for detecting glial and neuronal injury in mild TBI and have been described as the first marketed blood-based biomarker test in that setting (46). Related work has also evaluated serum UCH-L1 and GFAP in patients with acute stroke, supporting the broader concept that blood biomarkers can capture tissue-injury patterns relevant to stroke pathophysiology (47). Although these markers are not yet validated endpoints for selecting neuroprotective agents in AIS, they could serve as exploratory pharmacodynamic or enrichment biomarkers in future edaravone, EDB, or NBP trials, particularly when combined with neuroimaging and clinical risk profiles.

### 5.2 Adjunctive neuroprotective strategies in the era of endovascular therapy

EVT is standard care for eligible large-vessel occlusion, and adjunctive neuroprotection remains an active research question. TASTE-2 tested EDB before EVT and found a modest difference in 90-day functional independence in its trial population (7, 8). Observational studies of EDB and NBP during EVT, together with a Japanese database study of edaravone, are supportive but vulnerable to confounding (17, 18, 23). The phase II trial in patients with sufficient recanalization did not meet its primary 90-day functional endpoint for EDB (14). Thus, current data support further targeted trials rather than broad perioperative use of any agent; imaging, reperfusion status, and baseline injury burden are reasonable variables for prospective assessment.

### 5.3 Combination therapy with intravenous thrombolysis: current evidence

Intravenous thrombolysis is time-sensitive, and adjunctive neuroprotective regimens should be assessed as combination strategies rather than presumed synergistic treatments. The Bayesian NMA of rt-PA combinations reported mainly two-week NIHSS and Barthel Index outcomes and relied substantially on indirect comparisons and SUCRA rankings (27). The analysis included EDB plus rt-PA, edaravone plus rt-PA, and NBP plus rt-PA. Smaller studies and a meta-analysis suggest possible short-term benefit with edaravone plus alteplase, whereas NBP data remain preliminary (19, 20, 24). These data are insufficient to support routine protocolized integration or claims of pharmacological synergy.

### 5.4 Exploratory applications in special populations and stroke subtypes

Evidence in special populations and stroke subtypes remains limited. A retrospective posterior-circulation EVT study evaluated EDB and should be interpreted cautiously because of its single-center observational design (13). The phase II EDB study after successful reperfusion and observational reports of edaravone or NBP generate hypotheses about effect modification by recanalization status, age, or metabolic comorbidity, but do not establish subgroup-specific treatment recommendations (14, 17, 23, 25). Future trials should prespecify these subgroups and use consistent 90-day functional and safety endpoints.

## 6 Advances in the development of next-generation neuroprotective agents

### 6.1 Structural optimization of core drugs and development of new molecular entities

Medicinal chemists are pursuing next-generation neuroprotective candidates through rational structural optimization and molecular hybridization. These strategies aim to enhance pharmacological potency, improve pharmacokinetic profiles, including bioavailability and blood–brain barrier (BBB) permeability, and broaden target engagement. Such efforts seek to address limitations of current drugs while introducing new biological functions.

Preclinical studies have explored edaravone optimization. Esterification with borneol yielded pyrazolecarboxylic acid borneol ester derivatives; among these, candidate B16 showed greater free-radical-scavenging activity than edaravone, enhanced BBB penetration, rapid brain distribution, and reduced cerebral infarct volume in experimental models (48).

A complementary strategy involves designing multifunctional hybrid molecules. For example, integrating the edaravone scaffold with antiplatelet pharmacophores has generated dual-acting compounds with neuroprotective and antithrombotic activity, enabling multi-stage intervention in ischemic stroke pathophysiology and potentially reducing hemorrhagic risk (49, 50).

Further innovation includes novel N-salicylamide derivatives such as compound M11, a preclinical candidate reported to have neuroprotective activity and an improved safety margin in experimental models (51). Its relevance to clinical AIS treatment remains to be established.

Structural modification of NBP has also yielded candidate hybrids. Covalent fusion with ligustrazine produced compounds S8i and 7 m, which showed greater activity than NBP in cellular and animal models. Their mechanisms involved either Keap1-Nrf2 pathway activation or modulation of mitochondrial apoptosis signaling (52, 53).

Additionally, NBP has been repurposed as a scaffold for sulfide-donating prodrugs (54) and for 1,3,4-oxadiazole derivatives with antiplatelet activity (55). These innovations expand the therapeutic potential of parent compounds and may inform future multi-target anti-stroke drug development.

### 6.2 Innovations in targeted delivery systems: nanotechnology and novel delivery routes

Precise and efficient delivery of drugs to ischemic brain regions remains a major challenge in neuroprotective therapy. Nanotechnology-based delivery platforms and alternative administration routes are being investigated to address this challenge.

Nanocarrier systems may enhance the therapeutic index of neuroprotective agents through targeted surface modification and stimuli-responsive drug release. For example, a reactive oxygen species (ROS)-responsive nanocarrier encapsulating edaravone has been developed. It selectively releases the drug in response to elevated oxidative stress in the ischemic penumbra, potentially improving lesion-specific targeting, therapeutic efficacy, and safety (56).

Another approach is an NBP-based nanotherapeutic system engineered for triple targeting of endothelial cells, neurons, and microglia. This system undergoes dynamic morphological transformation within the pathological brain microenvironment, enabling spatiotemporally controlled drug release and concurrent modulation of the local milieu; it showed neuroprotection at reduced doses in preclinical studies (57).

Biomimetic strategies, including surface coating with platelet or macrophage membranes (58, 59) and receptor-mediated transcytosis, such as transferrin receptor-targeted transport across the blood–brain barrier (60), have improved brain accumulation of nanomedicines. These platforms may enhance targeting precision and support imaging-guided theranostics (58, 60).

Beyond intravenous delivery, alternative routes are being explored to circumvent the blood–brain barrier. Intranasal administration, which uses the direct nose-to-brain pathway, has attracted interest. Teng et al. (61) formulated a thermosensitive intranasal hydrogel co-loaded with edaravone and borneol. After nasal instillation, the formulation undergoes a sol–gel transition, prolonging mucosal residence and facilitating direct brain delivery through olfactory and trigeminal nerve pathways. In preclinical models, this approach bypassed the blood–brain barrier and hepatic first-pass metabolism and showed neuroprotective effects.

Intra-arterial delivery is another potential strategy for localized intervention. In an animal study, a single intra-arterial injection of edaravone combined with dexborneol conferred neuroprotection at low systemic doses, which was attributed to local anti-inflammatory and free-radical-scavenging activity (33).

Finally, prodrug design and advanced oral formulations, such as the brain-targeted NBP prodrug (62, 63), the self-nanomicellizing oral formulation of edaravone (64), and its ionic liquid derivative (65), may improve patient adherence and support chronic outpatient neuroprotective therapy.

## 7 Discussion and conclusion

Reperfusion therapy is established care for acute ischemic stroke, but the role of adjunctive neuroprotection remains to be defined. EDB, NBP, and edaravone each have a biological rationale and an evolving but unequal clinical evidence base. TASTE and TASTE-SL provide phase III evidence for EDB in broader AIS populations treated within 48 h, whereas TASTE-2 provides randomized evidence for EDB administered before EVT. BAST provides randomized evidence for NBP in patients receiving intravenous thrombolysis and/or EVT. The TASTE and TASTE-SL populations were not designed to establish efficacy in contemporary EVT-treated patients; therefore, their results should be interpreted separately from TASTE-2. Edaravone is supported mainly by observational studies and heterogeneous meta-analyses in contemporary reperfusion settings. These data do not demonstrate complementary clinical benefits among the agents, definitive efficacy across settings, or a basis for selection by recovery stage. Network meta-analysis rankings are useful for hypothesis generation but cannot substitute for direct comparisons with harmonized endpoints.

Mechanistic research links these agents to antioxidant, anti-inflammatory, mitochondrial, autophagy, apoptosis, and neurovascular pathways. Several pathways overlap among agents and injury models. Interpretation of individual mechanistic studies should consider the formulation tested, particularly EDB versus edaravone, and mechanistic observations should inform future trial hypotheses rather than be presented as proof of agent-specific clinical efficacy.

Future AIS trials should prospectively test whether biomarker profiles, imaging features, stroke subtype, reperfusion status, and comorbidities identify patients more likely to benefit from a given adjunctive neuroprotective regimen. GFAP, UCH-L1, oxidative-stress markers, and mitochondrial-injury readouts are reasonable exploratory pharmacodynamic or enrichment endpoints, but none is validated for clinical selection of edaravone, EDB, or NBP in AIS (46, 47). Structural optimization and targeted delivery platforms remain preclinical opportunities that require rigorous safety and efficacy evaluation before clinical adoption.

Rigorous, adequately powered trials with transparent eligibility criteria, standardized 90-day functional and safety outcomes, and direct comparative designs are needed to define whether, when, and for whom adjunctive neuroprotection improves outcomes after AIS reperfusion therapy.

## Appendix 1: Database-specific search strategies

PubMed/MEDLINE (last searched 31 May 2026): ((“Stroke”[Mesh] OR “Brain Ischemia”[Mesh] OR “acute ischemic stroke”[tiab] OR “cerebral infarction”[tiab]) AND (edaravone[tiab] OR “edaravone dexborneol”[tiab] OR dexborneol[tiab] OR butylphthalide[tiab] OR “DL-3-n-butylphthalide”[tiab] OR NBP[tiab])) AND (reperfusion[tiab] OR “endovascular thrombectomy”[tiab] OR thrombectomy[tiab] OR “intravenous thrombolysis”[tiab] OR alteplase[tiab] OR tenecteplase[tiab] OR “tissue plasminogen activator”[tiab]).

Embase (Elsevier): (‘brain ischemia’/exp. OR ‘ischemic stroke’:ti,ab,kw OR ‘acute ischemic stroke’:ti,ab,kw OR ‘cerebral infarction’:ti,ab,kw) AND (edaravone/exp. OR edaravone:ti,ab,kw OR ‘edaravone dexborneol’:ti,ab,kw OR dexborneol:ti,ab,kw OR butylphthalide:ti,ab,kw OR ‘dl 3 n butylphthalide’:ti,ab,kw OR nbp:ti,ab,kw) AND (reperfusion:ti,ab,kw OR ‘endovascular thrombectomy’:ti,ab,kw OR thrombectomy:ti,ab,kw OR ‘intravenous thrombolysis’:ti,ab,kw OR alteplase:ti,ab,kw OR tenecteplase:ti,ab,kw).

Web of Science Core Collection: TS = ((“acute ischemic stroke” OR “ischemic stroke” OR “cerebral infarction”) AND (edaravone OR “edaravone dexborneol” OR dexborneol OR butylphthalide OR “DL-3-n-butylphthalide” OR NBP) AND (reperfusion OR “endovascular thrombectomy” OR thrombectomy OR “intravenous thrombolysis” OR alteplase OR tenecteplase)).

Scopus: TITLE-ABS-KEY((“acute ischemic stroke” OR “ischemic stroke” OR “cerebral infarction”) AND (edaravone OR “edaravone dexborneol” OR dexborneol OR butylphthalide OR “DL-3-n-butylphthalide” OR NBP) AND (reperfusion OR “endovascular thrombectomy” OR thrombectomy OR “intravenous thrombolysis” OR alteplase OR tenecteplase)).

Cochrane Library: (“acute ischemic stroke” OR “ischemic stroke” OR “cerebral infarction”):ti,ab,kw AND (edaravone OR “edaravone dexborneol” OR dexborneol OR butylphthalide OR “DL-3-n-butylphthalide” OR NBP):ti,ab,kw.

Google Scholar: “acute ischemic stroke” (edaravone OR “edaravone dexborneol” OR dexborneol OR butylphthalide OR “DL-3-n-butylphthalide”) (thrombectomy OR thrombolysis OR alteplase), screened in relevance order.
