# Supplementary material for: Adjuvant neuroprotective therapies for acute ischemic stroke in the reperfusion era: a comparative narrative review of clinical evidence for edaravone, edaravone-dexborneol, and butylphthalide
Source: Front Neurol. 2026 Aug 12;17:1899933. doi: 10.3389/fneur.2026.1899933 (PMC13506449; doi:10.3389/fneur.2026.1899933)
Supplement: Supplementary file 1 [file Supplementary_file_1.docx]

# Supplementary Material — Literature Search Strategies

# Database: PubMed (National Library of Medicine)

Coverage: Inception through May 31, 2026

(

("Stroke"[Mesh] OR "Brain Ischemia"[Mesh] OR "acute ischemic stroke"[tiab] OR "cerebral infarction"[tiab])

AND

(edaravone[tiab] OR "edaravone dexborneol"[tiab] OR dexborneol[tiab] OR butylphthalide[tiab] OR "DL-3-n-butylphthalide"[tiab] OR NBP[tiab]))

AND

(reperfusion[tiab] OR "endovascular thrombectomy"[tiab] OR thrombectomy[tiab] OR "intravenous thrombolysis"[tiab] OR alteplase[tiab] OR tenecteplase[tiab] OR "tissue plasminogen activator"[tiab]).

)

# Database: Embase (Elsevier, via embase.com)

Coverage: Inception through May 31, 2026

('brain ischemia'/exp OR 'ischemic stroke':ti,ab,kw OR 'acute ischemic stroke':ti,ab,kw OR 'cerebral infarction':ti,ab,kw)

AND

(edaravone/exp OR edaravone:ti,ab,kw OR 'edaravone dexborneol':ti,ab,kw OR dexborneol:ti,ab,kw OR butylphthalide:ti,ab,kw OR 'dl 3 n butylphthalide':ti,ab,kw OR nbp:ti,ab,kw)

AND

(reperfusion:ti,ab,kw OR 'endovascular thrombectomy':ti,ab,kw OR thrombectomy:ti,ab,kw OR 'intravenous thrombolysis':ti,ab,kw OR alteplase:ti,ab,kw OR tenecteplase:ti,ab,kw).

# Database: Web of Science Core Collection (Clarivate)

Coverage: Inception through May 31, 2026

TS=(("acute ischemic stroke" OR "ischemic stroke" OR "cerebral infarction")

AND

(edaravone OR "edaravone dexborneol" OR dexborneol OR butylphthalide OR "DL-3-n-butylphthalide" OR NBP)

AND

(reperfusion OR "endovascular thrombectomy" OR thrombectomy OR "intravenous thrombolysis" OR alteplase OR tenecteplase)).

# Database: Scopus

Coverage: Inception through May 31, 2026

TITLE-ABS-KEY

(("acute ischemic stroke" OR "ischemic stroke" OR "cerebral infarction")

AND

(edaravone OR "edaravone dexborneol" OR dexborneol OR butylphthalide OR "DL-3-n-butylphthalide" OR NBP)

AND

(reperfusion OR "endovascular thrombectomy" OR thrombectomy OR "intravenous thrombolysis" OR alteplase OR tenecteplase)).

# Database: Cochrane Library

Coverage: Inception through May 31, 2026

("acute ischemic stroke" OR "ischemic stroke" OR "cerebral infarction"):ti,ab,kw

AND

(edaravone OR "edaravone dexborneol" OR dexborneol OR butylphthalide OR "DL-3-n-butylphthalide" OR NBP):ti,ab,kw.

# Database: Google Scholar

Coverage: Inception through May 31, 2026

"acute ischemic stroke" (edaravone OR "edaravone dexborneol" OR dexborneol OR butylphthalide OR "DL-3-n-butylphthalide") (thrombectomy OR thrombolysis OR alteplase), screened in relevance order. Mechanistic and biomarker searches added oxidative stress, neuroinflammation, mitochondrial dysfunction, GFAP, and UCH-L1 to the drug and AIS blocks.
